# Ethnic origin of Israeli Jews and psychological responses to the extreme stress of the ongoing war with Hamas in Gaza

**DOI:** 10.3389/fpsyg.2024.1403132

**Published:** 2024-10-23

**Authors:** Yohanan Eshel, Shaul Kimhi, Hadas Marciano, Bruria Adini

**Affiliations:** ^1^Stress and Resilience Research Center, Tel-Hai College, Tel Hai, Israel; ^2^Emergency & Disaster Management Department + ResWell Research Collaboration, School of Public Health, Faculty of Medical & Health Sciences, Tel Aviv University, Tel Aviv, Israel

**Keywords:** ethnic origin, extreme stress, psychologic responses, war, minority

## Abstract

**Introduction:**

Discrimination constitutes a source of stress for minority groups, leading to heightened levels of depression. Discrimination can also elicit positive responses aimed at reducing detrimental impacts. The stress experienced by minority groups may impact their mindset and influence their negative emotional responses. Israeli Jewish society comprised for many years two large communities: the dominant Ashkenazi people, who emigrated from East Europe, and the Mizrahi discriminated minority whose members came from Muslim countries. The Mizrahi minority has become a mainstream community over time, and its size equals the Ashkenazi group. This change raises an interesting issue that has not been investigated empirically: What characterizes the psychological responses to the stress of a formerly discriminated minority?

**Methods:**

Two representative samples responded to a similar questionnaire measuring inhibiting and bolstering coping strategies. The first sample of 930 people participated in this structured survey between October 12 and 19, 2022. No external adversity threatened Israel at that time. The second sample of 1,608 Israeli Jews participated between October 11 and 17, 2023, a few days after Hamas attacked the southern region of Israel, killed more than a 1,000 people, and kidnapped 100. We examine the impact of moderate and extreme stress of war on the maladaptive levels of anxiety and depression. Furthermore, we investigated the shielding psychological coping measures of this former minority, as compared to Ashkenazi group’s response.

**Results:**

Results show that the formerly minority Mizrahi group expresses higher levels of depression, anxiety, and sense of danger under extreme stress compared to the Ashkenazi group. Mizrahi individuals compensated concurrently for these negative emotions by fostering enhanced hope and societal resilience compared to the other group.

**Discussion:**

The main contributions of the present study are (a) Demonstration that psychological responses of descendants of a minority group to highly stressful conditions do not necessarily agree with their current status as a mainstream community. (b) Indicating that the phenomenon of concurrently enhanced negative and positive responses in face of extreme stress is associated with ethnic origin and history. (c) Associating the effects of different socio-demographic variables with the psychological response of the investigated groups to extreme and moderate stress.

## Introduction

### Ethnic identities of Israeli Jews

Israel is a land of immigrants. Its Jewish population grew about 10-fold during its seven decades of statehood due to incoming immigration. These migrants regarded Israel as their historical homeland, shared a belief in a common ancestry and a common religion, and currently share the Hebrew language (Lewin-Epstein and Cohen, 2019). Despite this similarity, they constitute two distinct communities: the dominant and veteran Ashkenazi people, most of whom emigrated from East Europe, and the Mizrahi people, who originated in the Muslim countries of the Middle East and North Africa. Fischer (2016) claims that the distinct identities of these communities reflect the different processes of modernization they experienced and their desired image of Israeli society. Most of the majority Ashkenazi people are secular individuals who wish to live in a universal, civil, liberal nation-state. The Mizrahi community consists mainly of religious people who strive to create an alternative Jewish society based on a vision of a primordial, traditional ethnic collectivity. Lewin-Epstein and Cohen (2019) assert that the stronger affinity of Mizrahi, especially the socioeconomically disadvantaged, with Jewish rather than Israeli identity indicates that many still feel somewhat excluded from the Zionist nation-building scheme devised by the Ashkenazi founders of Israel. The exclusion of Mizrahi Jews from full participation in the Zionist Israeli project, constructed by Ashkenazi Jews, and their sense of deprivation led to their forming a Mizrahi counter-collective (Fischer, 2016). Moreso, it contributed to the Mizrahi public’s tendency to vote for right-wing nationalist parties (Mizrachi, 2016; Peled and Shamir, 1990).

The founders of the State of Israel endorsed a strong ideology of integration of the exiles, made efforts to promote a mutual Israeli identity (Buzaglo, 2008) and molded the Mizrahi group according to their own cultural norms and values (Segev, 1986). The efforts to advance these national aims could not suppress the tendency of large communities to retain their traditional characteristics (Smooha, 2004). Lewin-Epstein and Cohen (2019) have found that ethnic identities resist change. The preference of the Mizrahi group to preserve its ethnic identity has slowed the efforts to acculturate it. Two separate Israeli Jewish identities evolved: an Ashkenazi identity that characterized the Jews of European origin and a Mizrahi identity endorsed by Jews from the Arab countries (Fischer, 2016; Smooha, 2004). Scholars pointed out several factors that enhanced this inter-group divergence: institutional discrimination (Shenhav, 2006), inequality of resources and socioeconomic attainments along the ethnic divide (Perlmann and Elmelech, 2012), educational and labor market disparities (Haberfeld and Cohen, 2007), and lower access to occupational opportunities experienced by the Mizrahi group (Lewin-Epstein et al., 1997).

Hobfoll’s (2010) conservation of resources theory posits that people strive to obtain, maintain, and protect things they value, such as social standing or a sense of mastery or power. People sense stress when their resources are threatened or lost, their achievements are not appreciated enough, and their efforts do not result in an adequate outcome. It seems that some of these issues bother parts of the Mizrahi group. Cohen et al. (2019) claim that ethnic inequalities and cleavage between the two Jewish populations continue in the second and the third generation: Mizrahi men of the third generation are less likely to hold academic degrees than second-generation Ashkenazi individuals. Labor market inequalities along the ethnic divide persist in the second generation (Haberfeld and Cohen, 2007) and even in the third generation (Cohen and Lewin-Epstein, 2018). There is reason to believe, therefore, that the continuous integration process of the Mizrahi community into Israeli society has left some of its members and their descendants with some sense of refutation, discrimination, and deprivation.

### Discrimination and psychological health

The social stress theory posits that perceived discrimination is a stressor adversely related to a broad range of physical and mental health outcomes (Anderson, 2013; Chen and Yang, 2014). Clinical studies of discriminated minority groups tend to agree that discrimination and deprivation are associated with maladaptive psychological outcomes and trigger psychological stress responses (Hatzenbuehler et al., 2013; Kessler et al., 1999). Being discriminated against often results in depressive symptoms (Alvarez-Galvez and Rojas-Garcia, 2019; Daoud et al., 2019). While overt expressions of discrimination have substantially decreased, certain groups continue to experience in recent decades, more nuanced and subtle forms of bias and prejudice (Pascoe and Richman, 2009). The impact of these subtle manifestations of discrimination on minority groups is at least as impactful as formal discrimination (Jones et al., 2016). Research shows accordingly that perceived discrimination increases the predisposition to suffer from mental symptoms such as anxiety, phobias, and depression (Everett et al., 2016).

Most studies on discrimination have traditionally emphasized its negative impacts on the psychological health of deprived minority groups. Crocker and Major (1989) posit that individuals from marginalized, stigmatized, and discriminated groups may internalize these adverse attitudes, leading to diminished self-esteem. They can also employ protective strategies to mitigate the adverse consequences of prejudice on mental well-being: (a) Refuting prejudices against their group. (b) Comparing their outcomes with members of their in-group rather than the out-group. (c) Emphasizing the attributes on which their group excels and devaluing the attributes on which their group typically fares poorly.

We posit that the response of large ethnic sections of society to subtle or implied discrimination can affect their self-assertion in both negative and positive ways. Such groups will be more vulnerable to extreme stress and regard it as more taxing and exceeding individual resources, as compared to other social groups that do not experience discrimination. This stress will intensify their maladaptive emotional response compared to other major segments of society. Moderate stress conditions often characterize the Israeli experience (Eran-Jona et al., 2022) and do not call for extreme emotional responses. Members of a community that senses discrimination, much the same as other social communities, will express neither high depression and anxiety nor high resilience and hope in response to moderate stress. In the face of highly distressing conditions, members of a group that regards itself as discriminated against would express higher depression, anxiety, and a sense of danger compared with other main sections of this society. In line with the self-protective properties of the stigma model (Crocker and Major, 1989) and the shielding self-esteem measures employed by discriminated minority groups (Fibbi et al., 2021), they would alleviate these harsh feelings. They will also compensate for them by high coping-supporting responses such as hope and patriotism. Other studies confirm the finding that the stress-buffering function of positive affect is more pronounced in individuals who experience high levels of stress (Okely et al., 2017; Pérez-Aranda et al., 2019).

### Stress and emotional expression

Lazarus (1991) posits that stress is a highly individual concept resulting from a person-environment transaction. The experience of stress and the ensuing emotional reactions differ significantly between individuals depending on how they interpret and appraise an occurrence: The emotional response to stressful events reflects the individual appraisal of whether the situation is one of stress, harm, loss, or challenge; whether the individual has the resources to manage this stress; and an evaluation of potential coping strategies. Appraisals are not necessarily conscious and may occur unconsciously at an automatic level (Jamieson et al., 2018). Stress and emotion retain a reciprocal dynamic relationship. Just as emotion determines the appraisal of an encounter, the stressful condition determines the individual’s emotional state. A study of the interactive mechanisms underlying perceived stress and its related negative emotions in everyday life (Feng et al., 2023) shows that daily perceived stress and negative emotions (i.e., perceived depression and anxiety) of students could reciprocally reinforce one another. People who accept stressful conditions more positively tend to experience lower levels of distress. Focusing on negative emotions, denying and disengaging the stress, or seeking emotional or social support are likely to result in experiencing higher levels of stress (Kumanova and Karastoyanov, 2013).

Recent studies suggest that the Mizrahi community has grown throughout the years, equals the size of the Ashkenazi group, and has gained some social hegemony in Israeli society (Abutbul-Selinger, 2023). The present study addresses an issue that, as far as we know, has not been investigated empirically so far: What characterizes the psychological responses of a formerly discriminated minority community whose social status changed into a mainstream group? We assume that despite the improved social, educational, economic and political conditions of the second and third generation of the Mizrahi Jews in Israel, they will express higher levels of detrimental emotions in face of highly stressful conditions, compared with members of the Ashkenazi community.

We examine these issues by investigating the coping-supporting and the coping-suppressing responses of the Mizrahi and Ashkenazi ethnic groups in two different stress conditions: (a) Coping with the moderate stress of everyday life in Israel. (b) Coping with the extreme distress of the current war with Hamas in the Gaza Strip.

Ethnic groups share other demographic characteristics, such as economic status, level of education, age, and gender, which may influence their resilience-supporting and suppressing responses in the face of highly stressful conditions. Rawal (2008) indicates that people characterized by low income, low level of education, younger age, and being women share high levels of social insecurity. In the context of the COVID-19 pandemic, the social insecurity of these groups significantly predicted their depression, anxiety, sense of danger, and societal resilience (Eshel et al., 2022). Controlling for such socio-demographic factors is essential for assessing the impact of ethnic origin on these affective reactions (Everett et al., 2016). The concurrent examination of the effects of ethnic group, gender, age, economic condition, and level of education will indicate the extent to which ethnic origin has an independent contribution to predicting the coping-suppressing and coping-supporting variables.

Throughout the years, mixed Mizrahi-Ashkenazi intermarriage gave birth to a third group of mixed-origin individuals. Research indicates that in some cases, being a member of a mixed-origin group is not experienced as problematic (Apitzsch and Gunduz, 2012), whereas in other cases, it raises complex subjective negotiations over identity (Chong, 2013). Since there is practically no research concerning the psychological responses of the mixed Mizrahi-Ashkenazi group to stressful conditions and no evidence concerning their similarity to one of their parents or the other, we do not include this group in the present study. The Mizrahi and Ashkenazi groups are employed to investigate the impact of perceived or insinuated discrimination on the emotional responses of large sections of society under extreme stress.

### Coping suppressing indices

#### Distress symptoms

Psychological symptoms constitute the most common human response to threats and disasters. Two main distress symptoms are anxiety and depression (Cénat et al., 2020). Research has shown that individual resilience decreases the strength of these symptoms (Labragu, 2021).

#### A sense of danger

Threats and disasters often raise a sense of danger. A threat to the life or the dignity of the individual or significant others increases the sense of danger (Eshel and Kimhi, 2016).

### Coping supporting indicators

#### Societal resilience

Is a broad concept concerning the trust in the social sustainability, strength, and integrity of national institutions (e.g., government and parliament), faith in social solidarity, and patriotism (Ben-Dor et al., 2002). During the COVID-19 pandemic, societal resilience positively correlated with individual and community resilience, well-being, hope, and morale. It negatively correlated with distress levels and a sense of danger (Kimhi and Eshel, 2019).

#### Hope

Snyder (2002) defines hope as “a cognitive set based on a reciprocally derived sense of successful goal-directed determination (termed as agency) and planning of ways to meet goals (termed as pathways) (p. 571).” Marciano et al. (2022) have demonstrated that in case of danger, hope is the best predictor of the sense of well-being, individual and societal resilience, and indices of anxiety and depression.

The present study examines the following hypotheses:

1. Ethnic origin will significantly affect the level of the psychological indicators of coping with the extreme stress condition after controlling for the demographic variables of age, gender, income, and level of education. The Mizrahi group would sense significantly higher depression, anxiety, and a sense of danger in the face of such distress compared with the Ashkenazi group.
2. Under this extreme stress, and controlled by the four demographic variables, these harsh feelings of the Mizrahi public will be accompanied by expressions of hope, trust in the government, perceived cohesiveness of Israeli society, patriotism, and confidence in the national institutions that will significantly surpass those of the Ashkenazi group. Ethnic origin will have no significant effect on the investigated psychological variables in the moderate-stress conditions after controlling for the effects of the demographic variables. The Mizrahi and the Ashkenazi groups will express about the same levels of these variables.
3. Women, younger adults, individuals with lower levels of education, and low-income people will be differently affected by these highly stressful conditions: They will score higher on the coping suppressing indices (depression, anxiety, and a sense of danger), and lower on coping-supporting indicators (hope and the four societal resilience factors), compared to men, older people, the more educated and the more affluent.

## Method

### Data collection

The two samples of the present data were collected online by an internet panel company possessing a database of more than 65,000 residents representing the diverse sections of Israeli society.^1^ The first sample of 930 Israeli Jews completed a structured survey between October 12 and 19, 2022. The second sample of 1,608 Israeli Jews responded to the same questionnaire between October 11 and 17, 2023, during a war between Israel and Hamas in Gaza, that followed the severe terror attack that Hamas launched against Israel. In both cases, the sampling was based on a stratified approach, aligned with the Israeli Central Bureau of Statistics data, appropriately representing the varied groups of the Jewish population (regarding gender, age, and geographic dispersal). Online questionnaires were distributed to potential participants, and the data collection continued until they reached the required number. All respondents expressed their informed consent to participate in the study, and their anonymity was maintained. The Ethics Committee of Tel Aviv University approved this research design.

### Participants

Table 1 presents the characteristics of the respondents of the first and the second investigated samples. The respondents’ ages in the two investigated samples ranged from 18 to 84 years; Males and females were 44.9, 55.1, 50.9, and 49.1% in the first and second samples, respectively. About half of the participants (47.2 and 51.8%) in the first and the second samples, respectively, reported a family income lower than Israel’s average family income. Almost half of them (44.8 and 44.6% in the first and the second samples, respectively) were secular, and almost half of them (48.2 and 46.7%, respectively) in the first and the second samples had academic degrees. The extreme stress sample was divided into 48% Mizrahi and 52.0% Ashkenazi respondents. The parallel percentages in the moderate stress sample were 44.9% Mizrahi and 55.1% Ashkenazi.

**Table 1:** The demographic characteristics of the extreme stress and the moderate stress condition samples.

Variable		Extreme stress		Moderate stress	
		*N*	%	*N*	%
Age groups	18–25	259	16.1	117	12.6
	26–35	346	21.5	177	19.0
	36–45	323	20.1	192	20.7
	46–55	249	15.5	165	17.7
	56–65	254	15.8	159	17.1
	66+	177	11.0	120	12.9
Gender	Male	798	49.6	473	50.9
	Female	810	50.4	457	49.1
Education	Up to high school	375	23.3	222	23.8
	Semi-academic	458	28.5	273	29.5
	Academic	775	48.2	435	46.7
Family income	Below average	759	47.2	482	51.8
	Average	484	30.1	260	28.0
	Above average	365	22.7	188	20.2
Religiosity	Secular	720	44.8	415	44.6
	Traditional	496	30.8	299	32.2
	Religious	222	13.8	119	12.8
	Orthodox	170	10.6	97	10.4
Ethnic origin	Mizrahi	772	48.0	418	44.9
	Ashkenazi	836	52.0	512	55.1

### Tools

#### Distress symptoms

Eight items of the (Derogatis and Savitz, 2000) BSI scale were employed: three on anxiety. (Example: ‘Feeling nervous’), and five on depression. (Example: ‘I see no hope for the future’). The response scale ranges from (1 = not at all) to (5 = to a very large extent). Respondents reported how much they currently experience any of the listed problems. The Cronbach’s alpha reliabilities of the anxiety scale in the first and the second samples were (*α* = 0.85) and (*α* = 0.88) respectively. The reliabilities for the depression scale in these samples were (*α* = 0.82) and (*α* = 0.83) respectively.

#### Sense of danger

The present study employed seven items of the sense-of-danger scale (Solomon and Prager, 1992). For example: ‘To what extent do you feel your life is in danger?’ The response scale ranges from (1 = not at all) to (5 = to a very large extent). The reliabilities of these scales in the higher and moderate stress conditions were (*α* = 0.83) and (*α* = 0.86), respectively.

#### Societal resilience

Kimhi and Eshel (2019) national resilience scale was employed. The four factors of this scale are (1) Trust in the government and its leaders. (Example: ‘I have confidence that the Israeli government will make the appropriate decisions in managing the upcoming crises’). (2) Social cohesion (solidarity) of Israeli society. (Example: ‘Israeli society enjoys a high social solidarity’). (3) A sense of attachment to the country: patriotism. (Example: ‘Israel is my home. I have no intention to leave it’). (4) Trust in the state institutions. (Example: ‘I trust the Israeli courts of law’). The response scale ranges from (1 = strongly disagree) to (6 = strongly agree). The present reliabilities of these scales in the higher and moderate-stress conditions, respectively, are (1) Trust in the government and its leaders: (*α* = 0.79) and (*α* = 0.79); (2) Social cohesion (solidarity): (*α* = 0.76) and (*α* = 77); (3) A sense of attachment to the country: Patriotism: (*α* = 0.80) and (*α* = 0.82); (4) Trust in the state institutions: (*α* = 0.70) and (*α* = 0.78).

#### Hope

The present 5-item hope scale derives from Jarymowicz and Bar-Tal (2006) scale. (Example: ‘I hope to emerge strengthened from the current crisis’). The response scale ranges from (1 = very little hope) to (5 = very high hope). The internal reliability of the scale in the higher and moderate stress conditions are (*α* = 0.93) and (*α* = 0.93) respectively.

#### Political attitudes

A single-item scale determined political views, ranging between (1. Extreme left) and (5. Extreme right).

#### Ethnic origin

A single item determined ethnic origin: “How do you define your origin group?” The response categories of the current study were: 1. Mizrahi, 2. Ashkenazi.

Four additional demographic characteristics were investigated: age, gender, income relative to the average income in Israel (1 = much below the average, 5 = much above the average), and education (1 = elementary school, 5 = academic degree).

## Results

Two sets of eight regression analyses examined the research hypotheses in the moderate and extreme-stress conditions and assessed the effects of ethnic origin on coping-suppressing and coping-supporting indicators. These effects were controlled for by the contributions of the following demographic variables: age, gender, income level, and level of education. The data presented in Table 2 support the investigated hypotheses. In agreement with hypothesis 1, ethnic origin significantly affected the three psychological indicators of coping-suppressing in extreme stress conditions after controlling for the effects of age, gender, income, and level of education. The Mizrahi group expressed significantly higher depression, anxiety, and a sense of danger in face of these distressing conditions compared with the Ashkenazi group. According to hypothesis 2, under such extreme stress, these harsh feelings of the Mizrahi public were accompanied by positive sentiments of hope, faith in the government and its leaders, the social solidarity of Israeli society, and a sense of attachment to the country (patriotism), that significantly surpassed these sentiments among the Ashkenazi group. Ethnic origin did not affect significantly the trust in the national institutions. In agreement with the second part of hypothesis 2, ethnic origin, controlled for the effects of the other demographic variables, was not significantly associated with seven of the eight psychological variables in the moderate-stress condition. Furthermore, the Mizrahi group expressed, in the moderate stress condition, a significantly lower sense of danger than the Ashkenazi public.

**Table 2:** Regression analyses for the effects of ethnic origin, gender, age, levels of income, and education, on psychological responses to extreme and moderate stress.

Predictors															
	Ethnic origin			Gender			Age			Income			Education		
Variables	*β*	*t*	*p*	*β*	*t*	*p*	*β*	*t*	*p*	*β*	*t*	*p*	*β*	*t*	*p*
Extreme stress															
Depression	−0.086	−3.522	0.000	0.156	6.361	0.000	−0.178	−7.192	0.000	−0.014	−0.533	0.594	0.051	1.987	0.047
Anxiety	−099	−4.159	0.000	0.215	9.039	0.000	−0.230	−9.522	0.000	0.041	1.675	0.094	0.058	2.330	0.020
Danger	−088	−3.580	0.000	0.206	8.412	0.000	−0.080	−3.225	0.001	−0.070	−2.770	0.006	0.044	1.733	0.083
F1	−0.133	−5.369	0.000	−0.124	−5.006	0.000	0.064	2.550	0.011	0.020	0.759	0.448	−0.122	−4.719	0.000
F2	−0.092	−3.664	0.000	0.022	0.864	0.388	−0.100	−3.970	0.000	0.008	0.324	0.746	−0.095	−3.634	0.000
F3	−0.082	3.275	0.000	−0.075	−3.021	0.003	0.119	4.696	0.000	−0.011	−0.436	0.663	−0.119	−4.574	0.000
F4	−0.048	−1.937	0.053	0.044	1.773	0.076	0.173	6.892	0.000	0.123	4.775	0.000	−0.088	−3.402	0.001
Hope	−0.071	−2.796	0.005	−0.074	−2.942	0.003	−0.048	−1.871	0.062	0.024	0.902	0.367	−0.072	−2.736	0.006
Moderate stress															
Depression	0.054	1.024	0.307	−0.152	−2.884	0.004	−0.019	−0.361	0.718	0.063	1.104	0.271	−0.110	−1.925	0.055
Anxiety	0.074	1.387	0.166	−0.065	−1.232	0.219	−0.109	−2.044	0.042	0.036	0.627	0.531	−0.061	−1.059	0.290
Danger	0.104	1.976	0.049	−0.028	−0.537	0.592	−0.129	−2.429	0.016	−0.111	−1.944	0.053	−0.009	−0.159	0.874
F1	0.024	0.470	0.638	0.039	0.758	0.449	0.285	5.535	0.000	0.119	2.146	0.033	−0.042	−0.766	0.444
F2	0.059	1.129	0.260	0.044	0.839	0.402	0.054	1.015	0.311	0.203	3.562	0.000	−0.159	−2.799	0.005
F3	0.088	1.693	0.091	0.054	1.035	0.301	0.222	4.244	0.000	0.055	0.986	0.325	−0.067	−1.185	0.237
F4	−0.014	−0.277	0.782	0.102	2.055	0.041	0.298	5.947	0.000	0.130	2.405	0.017	0.068	1.268	0.205
Hope	−0.023	−0.438	0.662	0.044	0.817	0.414	0.047	0.867	0.386	−0.011	−0.196	0.845	0.039	0.672	0.502

Gray values: significant results (*p*<0.05).

Hypothesis 3 claimed that women, younger adults, and individuals with lower levels of education and low income will be differently affected by this highly stressful condition: They will score high on the coping-suppressing indices and low on coping-supporting indicators. Table 2 partly supports this hypothesis. Women and young men followed this response pattern consistently in high-stress conditions. They expressed higher depression, anxiety, and a sense of danger and tended to score lower on societal resilience factors and hope compared with men and older people. Individuals with lower education revealed consistency in the opposite direction: Compared with more highly educated people, they scored lower on depression and anxiety and higher on all the coping-supporting variables. The level of income was not consistently associated with these variables. Table 2 shows further that gender and level of education were hardly associated with the predicted variables in the moderate-stress condition. The low-income persons scored lower than more affluent individuals on the coping-supporting variables. Younger adults seemed to be the most distressed group, and their responses in the moderate-stress condition were quite similar to their high-stress responses.

## Discussion

Research indicates two opposing psychological responses of marginalized, stigmatized, and disadvantaged minority groups to discrimination. The first is enhanced psychological stress that impairs mental health (Hatzenbuehler et al., 2013; Williams et al., 2019) and increases depression (Daoud et al., 2019). The second response consists of protective strategies, such as emphasizing the attributes on which their group excels, that moderate the adverse consequences of prejudice on mental well-being (Crocker and Major, 1989). This stress-buffering function of positive affect is more pronounced in individuals who experience high levels of stress (Okely et al., 2017; Pérez-Aranda et al., 2019). The present study examines what characterizes the psychological responses of a formerly discriminated minority whose social status has changed into a mainstream group. Our data show that this new status does not obliterate the impact of continuous prejudice and deprivation, and members of such groups retain these two modes of coping with prejudice. These data show further that communities may feel discriminated against despite being a substantial part of society. An elevated sense of societal resilience and hope seems to provide some emotional support and alleviate the promoted anxiety and depression expressed by them in highly distressing conditions. Studies have thus shown that concurrent positive and negative emotions can emerge in the direst circumstances (Aspinwall and Macnamara, 2005).

The stress of a former minority due to discrimination is likely to be under control in moderate stressful conditions. Enhanced feelings of anxiety and depression would surface only in face of highly distressing conditions. The second and third post-immigration Mizrahi generations constitute a part of the Israeli mainstream and enjoy remarkable improvements in their social, economic, and political status compared to their parents. However, under the stress of war, they expressed significantly higher maladaptive psychological responses than their Ashkenazi counterparts. In the face of war, the Mizrahi group compensated for these distressing emotions by expressing concurrently enhanced societal resilience and hope. The members of the other group did not share these compensation measures.

The available empirical evidence refutes the idea that the present finding of combined high distress level and high hope and resilience reflects a free expression of emotions rather than feelings of deprivation. Members of the Mizrahi community share a collectivistic cultural background and have developed, throughout the years, a semi-traditional ethno-religious Israeli-Jewish collectivity (Fischer, 2016). Research shows that cultural norms of emotional expression vary across ethnic groups (Markus and Kitayama, 1991; Roseman et al., 1995) and regulate emotional responses (Butler et al., 2007). Members of collectivistic communities tend to utilize high emotion regulation strategies to modulate their affective experiences and prefer to reduce their open expression (Green et al., 2005; Weiss et al., 2022). Stress and emotions retain a reciprocal dynamic relationship. The present finding of the concurrent higher levels of negative and positive emotions probably indicates a higher level of experienced stress (Lazarus, 1991; Feng et al., 2023).

## Limitations

Three limitations of this study should be mentioned: First, we investigated two different samples. A better design should examine the present hypotheses using a longitudinal research design, in which the same sample would experience the two stress conditions. We believe, however, that the high reliability and validity of the measures employed support the results of this study. Second, the present data were collected online by an internet panel company whose database represents the various sections of Israeli society. Yet, this method provides no data about the response rates of any specific sub-group of the investigated samples. It does not indicate the reasons for not partaking of those who chose not to participate. Third, this study employs self-report questionnaires. Biased answers may impact its results. Despite these shortcomings, online methods of gathering national data for research using questionnaires are probably the most widespread sampling and data-collecting methods (Hays et al., 2015).

## Conclusion

The present findings point to four main conclusions:

1. The unique contribution of the present study is its demonstration that the psychological responses of descendants of a minority group to highly stressful conditions do not necessarily agree with its new status as a mainstream community.
2. Stress and emotion have a reciprocal dynamic relationship: a higher level of negative emotion indicates an assessment of the stress as more extreme, threatening, and resource-taxing (Lazarus, 1991; Folkman and Lazarus, 1988). The present study found that rather than being a general phenomenon, enhanced negative emotional responses in the face of highly stressful conditions are associated with ethnic origin.
3. Discriminated communities generally continue their lives and cope with difficulties and challenges despite being exposed to bigotry. Future research should go beyond these discriminated groups and look for additional negative indicators of dealing with perceived discrimination among other social units that experience subtler discrimination. It should also attempt to reveal additional positive psychological responses that constitute protective measures and co-exist with feelings of suppression and depression to counter prejudice.
4. Measures of coping-supporting, such as societal resilience, and coping-suppressing mechanisms, such as distress symptoms, are often employed as indicators of the state of a community or a nation in distressing conditions (Kimhi et al., 2021). The present findings emphasize that these indices are expressions of subjective perceptions of reality. These perceptions represent individual and group levels of fears and anxieties as well as psychological measures employed to overcome and defend against them. Assessing the state of any distressed community, using either coping-supporting or coping-suppressing indicators, may result in substantially positively or negatively biased conclusions when the sample employed does not represent the investigated population correctly.

## Data Availability

The original contributions presented in the study are included in the article/supplementary material, further inquiries can be directed to the corresponding author.

## Footnotes

^1^ https://sekernet.co.il/Index.aspx
